# Supplementary material for: The longitudinal relations between physical activity, inflammation, and depression in the Health and Retirement Study
Source: PLOS Ment Health. 2025 Apr 24;2(4):e0000211. doi: 10.1371/journal.pmen.0000211 (PMC12798416; doi:10.1371/journal.pmen.0000211)
Supplement: Attachment — Submitted filename: Response to Reviewers.docx [file pmen.0000211.s002.docx]

**Response to Reviewers**

We have included each comment from the journal/reviewers in block quotes followed by our response to each suggestion / comment.

**Journal Requirements:**

1. We noticed you have some minor occurrence of overlapping text with the following previous publication(s), which needs to be addressed:

- <https://doi.org/10.3389/fpsyg.2020.571943>
- doi:10.1016/j.psyneuen.2018.05.035.

In your revision ensure you cite all your sources (including your own works), and quote or rephrase any duplicated text outside the methods section. Further consideration is dependent on these concerns being addressed.

**Response:**

We deeply appreciate the concern of overlapping text with the referenced publication. After examining the similarities between the papers using iThenticate, we found that the overlapping text is coincidental due to overlapping RI-CLPM methods. We subsequently believe that there are no substantive similarities to address that would require rephrasing, quoting, or a citation. If we are missing other similarities please advise specifically as to what they may be and we will be more than happy to address them appropriately.

**Review Comments to the Author**

**Reviewer #1**

The manuscript entitled "The longitudinal relation between physical activity, inflammation, and depression: A mediational analysis using the Health and Retirement Study" has a very interesting topic. It is well written. However, references should be up date with more recent evidence and also its related discussion section.

**Response:**

We thank the reviewer for requesting more recent evidence to support the paper. Several more recent citations have been added throughout the Introduction and Discussion sections where appropriate to incorporate more recent evidence into the manuscript. Please see:

Guo X, Le Y. The triangular relationship of physical activity, depression, and inflammatory markers: A large cross-sectional analysis with NHANES data. J Affect Disord. 2024 Dec 15;367:589–97.

Tiagha RA, Eteneneng JE, Chungag BN. The effect of physical inactivity on inflammatory markers in the geriatric population: A systematic review. NILE j Geriatr Gerontol [Internet]. 2024 Oct 10; Available from: https://journals.ekb.eg/article_385376.html

Hernández-Lepe MA, Ortiz-Ortiz M, Hernández-Ontiveros DA, Mejía-Rangel MJ. Inflammatory profile of older adults in response to physical activity and diet supplementation: A systematic review. Int J Environ Res Public Health. 2023 Feb 25;20(5):4111.

Bautmans I, Salimans L, Njemini R, Beyer I, Lieten S, Liberman K. The effects of exercise interventions on the inflammatory profile of older adults: A systematic review of the recent literature. Exp Gerontol. 2021 Apr;146(111236):111236.

Zenebe Y, Akele B, W/Selassie M, Necho M. Prevalence and determinants of depression among old age: a systematic review and meta-analysis. Ann Gen Psychiatry. 2021 Dec 18;20(1):55.

Xu Y, Wang M, Chen D, Jiang X, Xiong Z. Inflammatory biomarkers in older adults with frailty: a systematic review and meta-analysis of cross-sectional studies. Aging Clin Exp Res. 2022 May 4;34(5):971–87.

Beavers DP, Kritchevsky SB, Gill TM, Ambrosius WT, Anton SD, Fielding RA, et al. Elevated IL-6 and CRP levels are associated with incident self-reported major mobility disability: A pooled analysis of older adults with slow gait speed. J Gerontol A Biol Sci Med Sci. 2021 Nov 15;76(12):2293–9.

Calder PC, Bosco N, Bourdet-Sicard R, Capuron L, Delzenne N, Doré J, et al. Health relevance of the modification of low grade inflammation in ageing (inflammageing) and the role of nutrition. Ageing Res Rev. 2017 Nov 1;40:95–119.

**Reviewer #2**

1. The Introduction may benefit from more precise identifications of literature gaps that the current investigation addresses. For example, the review of the effects of physical activity on inflammation (the paragraph starting on line 123) led to the conclusion that “more research is needed to investigate if physical activity reduces inflammation in depressed individuals”, leaving readers to expect the current investigation would target individuals with depression, but this isn’t the case. In a similar vein, the theoretical motivation for examining the associations among physical activity, inflammation, and depression in older adults is lacking. It is unclear why examining the current questions among older adults is important and why similar hypotheses, as expected by the reviewed evidence from younger populations, are expected in the current sample of older adults.

**Response to Comment #1**

We appreciate the feedback on tailoring the Introduction to better match the study design and have made appropriate changes in line with this feedback. Line 182 (previously line 123) has been altered to more generally emphasize the focus on studying depressive symptoms in the general population as opposed to clinically depressed adults. An additional paragraph has also been added (starting on line 184) to provide additional theoretical motivation for investigating the relations between physical activity, inflammation, and depressive symptoms in older adults. We also revised language throughout the paper to specify older adults and symptoms of depression where appropriate.

2. As the novel piece of the current investigation is between- vs. within-persons associations among physical activity, inflammation, and depression, the Introduction can benefit from theoretical reasons for this distinction (rather than simply that use of RI-CLPM allows for it). That is, what are we gaining from examining within-persons associations that we are not already gaining from the less resource-intensive investigations of between-persons associations as well as RCT that allows establishment of causality?

**Response to Comment #2**

We appreciate the reviewer’s comment highlighting the need to clarify the theoretical rationale for distinguishing between within- and between-person associations. As noted by [(Hamaker et al., 2015)](https://paperpile.com/c/gJYFqd/BNM02), the inability of traditional Cross-Lagged Panel Models (CLPM) to separate these effects can lead to biased or erroneous conclusions regarding the magnitude and direction of cross-lagged associations. By disaggregating these components, the Random Intercept Cross-Lagged Panel Model (RI-CLPM) allows us to isolate within-person dynamic processes while controlling for stable, trait-like differences at the between-person level. This distinction is critical for understanding whether observed associations reflect changes within individuals over time or stable differences between individuals.

The value of focusing on within-person associations lies in its relevance to causal mechanisms and potential interventions. While between-person analyses help identify general trends, within-person associations allow us to examine how deviations in physical activity, inflammation, and depression co-vary over time, potentially revealing actionable targets for clinical interventions. This approach complements randomized controlled trials (RCTs) by uncovering naturalistic patterns of change that may inform the design of experimental interventions. This additional rationale has been added to the manuscript’s introduction starting on line 209**.**

3. There are three major methodological concerns:
(a) Given the 3 timepoints of data, there are more fitting statistical methods for modeling within-individual changes. Currently, RI-CLPM models T1 T2 and then T2 T3, AND these links are constrained to be the same, so the 3 timepoints of data is not being utilized to adequately. Yet, both MLM and DSEM are statistical models that can estimate trajectories of depression/inflammation across all 3 timepoints. Particularly, DSEM will allow you to estimate autoregressive links across all three timepoints for all variables, lagged-associations, as well as the associations among within-individual trajectories of inflammation (time inflammation), trajectories of physical activity (time activity), and trajectories of depression (time depression), as well as how these activity trajectories may covary with depression trajectories mediated by inflammation trajectories. In my opinion, the use of RI-CLPM is not examining the kind of within-persons changes expected from data that has 3 datapoints (rather than 2).

**Response to Comment #3a**

Perhaps this reviewer isn’t aware that the random-intercept cross-lagged panel model (RI-CLPM) is considered a fully dynamic structural equation model [(e.g., Falkenström et al., 2022)](https://paperpile.com/c/gJYFqd/Yr1uT/?prefix=e.g.%2C). It can be confusing because dynamic SEM (DSEM) was developed for intensive longitudinal study designs with **many** observations from individuals over time. In other words, it combines time series analyses with structural equation modeling [(Asparouhov et al., 2018)](https://paperpile.com/c/gJYFqd/RQK6w). It is typically used when there are at least 10 or more repeated measures. For instance, in a comprehensive review of statistical power for DSEM models, involving one of the creators of DSEM, they did not consider power analyses for any simulated studies that had fewer than 10 within-person assessments [(Schultzberg & Muthén, 2018)](https://paperpile.com/c/gJYFqd/ycYwW) and found good statistical power when: a) N = 150 subjects and 25 within-person assessments, b) N = 75 subjects and 75 within-person assessments, and c) N = 50 subjects and 150 with-person assessments. These study designs are obviously quite different from the current study (with only 3 waves of data).

However, the random intercept cross-lagged panel model (RI-CLPM) is a DSEM explicitly developed to address situations where there are less than 10 repeated measurements. In fact, in the initial paper [(Hamaker et al., 2015)](https://paperpile.com/c/gJYFqd/BNM02), the developers of RI-CLPM used a study with three waves of data (“RI-CLPM requires at least three waves of data”, pg. 105) to illustrate the advantages of the RI-CLPM over the traditional CLPM [(see Figure 1 in Hamaker et al., 2015)](https://paperpile.com/c/gJYFqd/BNM02/?prefix=see%20Figure%201%20in). Among these advantages include the separate modeling of within-person change from stable, trait-like, between-person components, which is exactly what RI-CLPM is designed to do. Thus, we believe we have done what this reviewer suggested–used a dynamic SEM model to examine associations within these data.

Given this confusion about the RI-CLPM, we have now added additional text to the introduction (see line 206 and the paragraphs starting with line 209) that attempts to make it clearer that RI-CLPM is a form of dynamic SEM and allows researchers to examine auto-regressive and cross-lagged effects at the within-person level while simultaneously controlling for stable, trait-like effects at the between-person level.

(b) The way RI-CLPM is implemented raises some concerns. While important, model fits do not solely determine whether the model is appropriate for the research questions at hand. By nature of constraining the many estimates (grand mean, autoregressive, cross-lagged, and covariance structure all were constrained) model fit will increase merely because less is being asked from the model to estimate. But constraining those paths do not necessary make sense theoretically—e.g., grand means being constrained to equal requires the theoretical assumption that there’s no systematic change in the average level of inflammation, depression, and physical activity, but we know CRP increases with age in the U.S., depression increases in older adults, physical activity decreases in older adults; another example: constraining cross-lagged links to equal requires the theoretical assumption that, for example, the prediction of physical activity on CRP is static over time, despite expected aging-related physiological declines. Given all these constraints imposed, it’s unclear how to draw conclusions from the findings, yet the Discussion does not interpret the findings in light of the theoretical implication of these constraints.

**Response to Comment #3b**

We agree that one has to take into account the theoretical model underlying the data. One way to test a theoretical model is to place constraints on the model and see if it continues to fit the data well. For instance, placing constraints on the cross-lagged relationships between physical activity and CRP to be equal over time allows us to empirically test whether these associations differ over time (if they substantially differ, placing constraints should hurt the model performance). In this case, we did not find that model constraints hurt model performance, suggesting there were not strong differences in the cross-lagged associations over time. Keep in mind that these cross-lagged relationships are only concerned with change at the within-person level after controlling for the between-person, trait-like variance. Prior work examining the linkages between activity and CRP did not disentangle between- and within-person variance, and the stable trait-like variance could have been driving previously observed associations. Indeed, in the current study, we found strong associations for activity level and CRP at the between-subjects level but not at the within-subjects level.

Nevertheless, we agree that we should mention these constraints in the discussion and now discuss the implications of these constraints more fully than our original submission. Please see line 696 where we write:

“It was also notable that the model continued to fit well even after constraining the auto-regressive path for each variable and cross-lagged relationships between variables to be equal over time. Constraints on those associations did not hurt overall model fit, suggesting there were not strong differences in the strength of the cross-lagged associations over time. Prior work examining the linkages between physical activity and CRP did not disentangle the between- and within-person variance, so the stable trait-like variance could have been driving previously observed associations. Indeed, in the current study, we found strong associations for activity level and CRP at the between-subjects level but not at the within-subjects level.”

(c) There are somewhat inaccurate statements in describing within-persons associations in the context of RI-CLPM. As one example, in RI-CLPM, within-person associations refer to the cross-lagged associations, as you cannot really have within-person associations within a single wave, so statements like these “There were two significant but weak within-person associations within Wave 1 (Fig 2). A small negative within-person correlation existed between reported physical activity and reported CES-D scores at Wave 1 (β = -0.08, SE = 0.174, p < 0.001), indicating that within Wave 1 individuals who were more physically active tended to have a lower depression score, while those that were less active tended to have a higher depression score” (Line 359). The nature of these associations are more between-person, than within-person, associations, even if scores were person-mean centered.

**Response to Comment #3c**

Given that this reviewer wasn’t aware that we were using dynamic SEM, I can see how this reviewer might arrive at this conclusion. However, we took this opportunity to further clarify our measurement and now refer to the variables as within-subjects deviation scores, as this more accurately depicts what is measured (i.e., deviation from the mean at that time point).

We now write (line 495), There were two significant but weak within-person associations within Wave 1 (Fig 2). A small negative correlation existed for the physical activity deviation score and CES-D deviation score at Wave 1 (β = -0.08, *SE* = 0.174, p < 0.001), indicating that within Wave 1 individuals who were more physically active than average tended to have a lower depression score than average, while those that were less active than average tended to have a higher depression score than average. Additionally, contrary to what prior research might suggest, there was a small positive within-person correlation between reported physical activity deviation score and CRP deviation levels at Wave 1 (β = 0.06, *SE* = 0.109, p < 0.001), indicating that individuals who were more physically active than average tended to have higher CRP levels than average, and vice versa.”

4. HRS has other inflammatory cytokines (e.g., IL-6, TNF-a). Since the Introduction mentioned discrepant findings based on the marker assessed, would the authors consider parallel analyses with these cytokines?

**Response to Comment #4**

We thank the reviewer for noting the availability of IL-6 and TNF-α in the HRS and agree that, ideally, we could investigate those inflammatory biomarkers in addition to CRP. While we recognize the importance of exploring multiple inflammatory biomarkers, IL-6 and TNF-α were only introduced within HRS in 2014 and 2016, respectively, whereas CRP data spans 2006–2016. To maximize sample size and maintain consistency across waves, our current analysis focused on CRP. Future studies may incorporate IL-6 and TNF-α as more HRS data becomes available, potentially allowing for parallel analyses and deeper insights into biomarker-specific effects. This clarification has been incorporated into the Discussion section starting on line 716.

For more information on which biomarkers are available in each HRS wave, please see the most recent documentation of blood-based biomarkers in HRS [(Crimmins et al., 2020)](https://paperpile.com/c/gJYFqd/hoMOA).

5. The Discussion section largely explains the current null or opposite-than-hypothesized findings methodologically (e.g., 4 year lag might be too much to capture the more transient changes, sum score of physical activity obscuring findings). As described above, I agree there are methodological concerns, so along with the authors’ methodological interpretations of the findings, it does leave readers wondering whether the current methods are well-positioned to test the research questions authors set out to test from the get go. That said, I really think the examination of between- vs. within-individual associations is important, so would the authors consider using more fitting methods so that we have a better sense whether the unexpected/null results are due to suboptimal methods or in fact reflect the true nature of these relationships?

We appreciate the methodological concerns raised by this reviewer and hope that our responses to Comments #3a-c above justify our use of RI-CLPM in the current study.

6. Minor: Line 134 suggests that “physical activity led to decrease in both pro-inflammatory markers and depressive symptoms” but that CRP was not available in that study, please state the pro-inflammatory markers assessed.

The pro-inflammatory marker assessed in study was IL-6. This has been clarified in the paper on line 167 (previously line 134).

**Reviewer #3:**

The authors investigated the relationship between physical exercise and CRP and depression in elderly adults.They found a result in a cross-sectional study indicating that physical exercise reduces acute inflammation and depression scores. However, in a longitudinal study, they found no association between exercise, CRP, and depression. The authors discuss the limitations of the longitudinal study's long interval between assessment phases and possible differences between exercise protocols. It is also important to highlight that elderly adults may have low-grade chronic inflammation that involves other markers and chronic inflammatory mechanisms. These mechanisms may be more noticeable in chronic inflammation and, therefore, more related to the influence of exercise over time. The authors discussed and pointed out the strengths and weaknesses and made some suggestions.

I only suggest that the authors highlight that the individuals were elderly and, therefore, other markers could be more appropriate for assessing chronic inflammation, in addition to the lag between one phase and another throughout the study.

**Response to Reviewer #3**

We thank the reviewer for the insightful suggestion to highlight that the individuals in our study were elderly and to consider other markers more appropriate for assessing chronic inflammation in this population. We have revised the Introduction and Discussion section to explicitly acknowledge the unique characteristics of chronic inflammation in older adults and the potential utility of additional biomarkers, such as IL-6 and TNF-α, in capturing these processes. Specifically, we have added a paragraph in the Discussion section (see line 709) that discusses the concept of "inflammaging" and underscores the need for future research to incorporate a broader panel of biomarkers to provide a more comprehensive understanding of chronic inflammation in elderly populations. Also see the paragraph in the Introduction starting on line 184 in response to critique #1 from Reviewer #2.

**References**

[Asparouhov, T., Hamaker, E. L., & Muthén, B. (2018). Dynamic structural equation models. *Structural Equation Modeling: A Multidisciplinary Journal*, *25*(3), 359–388.](http://paperpile.com/b/gJYFqd/RQK6w)

[Crimmins, E., Faul, J., Kim, J. K., & Weir, D. (2020). Documentation of blood-based biomarkers in the 2016 Health and Retirement Study. *Ann Arbor, MI: University of Michigan, Survey Research Center*.](http://paperpile.com/b/gJYFqd/hoMOA) <https://hrsdata.isr.umich.edu/sites/default/files/documentation/data-descriptions/HRS%20Data%20Documentation%20for%202016%20DBS%20Release.pdf>

[Falkenström, F., Solomonov, N., & Rubel, J. A. (2022). How to model and interpret cross-lagged effects in psychotherapy mechanisms of change research: A comparison of multilevel and structural equation models. *Journal of Consulting and Clinical Psychology*, *90*(5), 446–458.](http://paperpile.com/b/gJYFqd/Yr1uT)

[Hamaker, E. L., Kuiper, R. M., & Grasman, R. P. P. P. (2015). A critique of the cross-lagged panel model. *Psychological Methods*, *20*(1), 102–116.](http://paperpile.com/b/gJYFqd/BNM02)

[Schultzberg, M., & Muthén, B. (2018). Number of subjects and time points needed for multilevel time-series analysis: A simulation study of dynamic structural equation modeling. *Structural Equation Modeling: A Multidisciplinary Journal*, *25*(4), 495–515.](http://paperpile.com/b/gJYFqd/ycYwW)
